# A polygenic risk score for nasopharyngeal carcinoma shows potential for risk stratification and personalized screening

**DOI:** 10.1038/s41467-022-29570-4

**Published:** 2022-04-12

**Authors:** Yong-Qiao He, Tong-Min Wang, Mingfang Ji, Zhi-Ming Mai, Minzhong Tang, Ruozheng Wang, Yifeng Zhou, Yuming Zheng, Ruowen Xiao, Dawei Yang, Ziyi Wu, Changmi Deng, Jiangbo Zhang, Wenqiong Xue, Siqi Dong, Jiyun Zhan, Yonglin Cai, Fugui Li, Biaohua Wu, Ying Liao, Ting Zhou, Meiqi Zheng, Yijing Jia, Danhua Li, Lianjing Cao, Leilei Yuan, Wenli Zhang, Luting Luo, Xiating Tong, Yanxia Wu, Xizhao Li, Peifen Zhang, Xiaohui Zheng, Shaodan Zhang, Yezhu Hu, Weiling Qin, Bisen Deng, Xuejun Liang, Peiwen Fan, Yaning Feng, Jia Song, Shang-Hang Xie, Ellen T. Chang, Zhe Zhang, Guangwu Huang, Miao Xu, Lin Feng, Guangfu Jin, Jinxin Bei, Sumei Cao, Qing Liu, Zisis Kozlakidis, Haiqiang Mai, Ying Sun, Jun Ma, Zhibin Hu, Jianjun Liu, Maria Li Lung, Hans-Olov Adami, Hongbing Shen, Weimin Ye, Tai-Hing Lam, Yi-Xin Zeng, Wei-Hua Jia

**Affiliations:** 1 State Key Laboratory of Oncology in South China, Collaborative Innovation Center for Cancer Medicine, Guangdong Key Laboratory of Nasopharyngeal Carcinoma Diagnosis and Therapy, Sun Yat-sen University Cancer Center, Guangzhou, P. R. China; 2 Cancer Research Institute of Zhongshan City, Zhongshan Hospital of Sun Yat-sen University, Zhongshan, China; 3 School of Public Health, The University of Hong Kong, Hong Kong S.A.R., China; 4 Center for Nasopharyngeal Carcinoma Research (CNPCR), The University of Hong Kong, Hong Kong S.A.R., China; 5 Radiation Epidemiology Branch, Division of Cancer Epidemiology and Genetics, National Cancer Institute, National Institutes of Health, Bethesda, MD USA; 6 Wuzhou Red Cross Hospital, Wuzhou, Guangxi P.R. China; 7 Wuzhou Cancer Center, Wuzhou, Guangxi P.R. China; 8 Key Laboratory of Cancer Immunotherapy and Radiotherapy, Chinese Academy of Medical Sciences, Ürümqi, Xinjiang Uygur Autonomous Region 830011 P.R. China; 9 Department of Genetics, Medical College of Soochow University, Suzhou, China; 10 School of Public Health, Sun Yat-sen University, Guangzhou, P.R. China; 11 Public Health Service Center of Xiaolan Town, Zhongshan City, Guangdong China; 12 State Key Laboratory of Pathogenesis, Prevention and Treatment of High Incidence Diseases in Central Asia, Departments of Institute for Cancer Research, The Third Affiliated Hospital of Xinjiang Medical University, Ürümqi, 830011 P.R. China; 13 Key Laboratory of Oncology of Xinjiang Uyghur Autonomous Region, Ürümqi, 830011 China; 14 Departments of Institute for Cancer Research, The Third Affiliated Teaching Hospital of Xinjiang Medical University, Affiliated Cancer Hospital, Ürümqi, Xinjiang Uyghur Autonomous Region 830010 P.R. China; 15 Center for Health Sciences, Exponent, Inc., Menlo Park, CA USA; 16 Stanford Cancer Institute, Stanford, CA USA; 17 Department of Otolaryngology-Head and Neck Surgery, First Affiliated Hospital of Guangxi Medical University, Nanning, Guangxi China; 18 Department of Epidemiology, International Joint Research Center on Environment and Human Health, Center for Global Health, Jiangsu Key Lab of Cancer Biomarkers, Prevention and Treatment, Collaborative Innovation Center for Cancer Medicine, Nanjing Medical University, Nanjing, China; 19 Division of Infection and Immunity, Faculty of Medical Sciences - University College London, London, UK; 20 International Agency for Research on Cancer (IARC/WHO), Lyon, France; 21 Department of Nasopharyngeal Carcinoma, Sun Yat-sen University Cancer Center; State Key Laboratory of Oncology in South China, Collaborative Innovation Center for Cancer Medicine, Sun Yat-sen University Cancer Center, Guangzhou, China; 22 Department of Radiation Oncology, Sun Yat-sen University Cancer Center, State Key Laboratory of Oncology in South China, Collaborative Innovation Center for Cancer Medicine, Guangdong Key Laboratory of Nasopharyngeal Carcinoma Diagnosis and Therapy, Guangzhou, China; 23 Human Genetics, Genome Institute of Singapore, Agency for Science, Technology and Research (A*STAR), Singapore, Singapore; 24 Department of Medicine, Yong Loo Lin School of Medicine, National University of Singapore, Singapore, Singapore; 25 Department of Clinical Oncology, The University of Hong Kong, Hong Kong S.A.R., China; 26 Clinical Effectiveness Group, Institute of Health and Society, University of Oslo, Oslo, Norway; 27 Department of Medical Epidemiology and Biostatistics, Karolinska Institutet, Stockholm, Sweden; 28 Department of Epidemiology and Health Statistics & Key Laboratory of Ministry of Education for Gastrointestinal Cancer, Fujian Medical University, Fuzhou, China

**Keywords:** Epidemiology, Cancer genetics, Predictive markers

## Abstract

Polygenic risk scores (PRS) have the potential to identify individuals at risk of diseases, optimizing treatment, and predicting survival outcomes. Here, we construct and validate a genome-wide association study (GWAS) derived PRS for nasopharyngeal carcinoma (NPC), using a multi-center study of six populations (6 059 NPC cases and 7 582 controls), and evaluate its utility in a nested case-control study. We show that the PRS enables effective identification of NPC high-risk individuals (AUC = 0.65) and improves the risk prediction with the PRS incremental deciles in each population (*P*_*trend*_ ranging from 2.79 × 10^−7^ to 4.79 × 10^−44^). By incorporating the PRS into EBV-serology-based NPC screening, the test’s positive predictive value (PPV) is increased from an average of 4.84% to 8.38% and 11.91% in the top 10% and 5% PRS, respectively. In summary, the GWAS-derived PRS, together with the EBV test, significantly improves NPC risk stratification and informs personalized screening.

## Abstract (web summary)

Polygenic Risk Scores (PRS) show promise for facilitating cancer risk stratification. Here, the authors performed a large genome-wide association study and develop a PRS for nasopharyngeal carcinoma, which could improve risk stratification and personalized screening.

## Introduction

Nasopharyngeal carcinoma (NPC) is one of the most common malignancies in East and Southeast Asia, where >70% of all 129,079 worldwide cases were diagnosed in 2018^1–3^. In endemic regions, NPC incidence peaks at the age of 40–65 years^4^. Nearly 80% of the NPC patients are diagnosed at an advanced stage^5^. Given the peak occurrence of NPC at a relatively young age and the poor prognosis, NPC contributes prominently to the cancer burden in endemic areas with substantial economic and societal impacts^6^.

However, the insufficient explanatory power of modifiable risk factors^7–9^ has hindered effective primary preventive strategies for NPC^10^. Because fewer than 10% of NPC patients present with stage I disease, when the 5-year overall survival rate is 90% or higher^11–13^, the emphasis has been on secondary prevention using screening to detect early, asymptomatic disease. Based on the close relationship between NPC and Epstein-Barr virus (EBV) infection, the anti-EBV IgA serological test has been recommended by the Chinese Ministry of Health^14^ and is widely used as a screening tool in China^15,16^. According to the current NPC screening strategy, individuals were recommended to be screened by two anti-EBV antibodies (VCA-IgA and EBNA1-IgA) between the ages of 30 and 69 years in NPC endemic areas. The high-risk individuals by the preliminary serological test were further recommended for clinical examinations, such as nasopharyngeal fiberscope, and even a pathological biopsy for additional confirmation when necessary. Our prospective NPC screening study showed that the anti-EBV IgA test could improve early diagnostic rate (79.0% for the screened participants versus 22.4% for the non-screened participants) and decrease NPC mortality (1.8 per 100 000 person-year for the screened participants versus 8.3 per 100,000 person-year for the non-participants)^12,17^. However, the positive predictive value (PPV) of the anti-EBV IgA test was only about 4%^12,15^. Consequently, >95% of subjects undergo unnecessary clinical examinations following a false-positive screening test^16^, which results in low compliance and screening efficiency. So, it is necessary to find a complementary method to improve the current screening strategy by avoiding unnecessary screening while keeping the power to identify high-risk individuals.

Recent large-scale population studies suggest that a polygenic risk score (PRS) that combines the effects of common genetic variants might be effectively used to identify individuals at high risk of complex diseases^18^. The low positive predictive value of the currently used EBV-based screening tool, coupled with the high heritability of NPC^19–26^, makes NPC an ideal candidate disease for the development of a PRS to facilitate risk stratification, especially in high-risk areas of southern China. As the PRS could be used as an indicator of an individual’s inherent genetic risk for developing the disease at various ages in his lifetime, it can be calculated long before the onset of disease and substantially guide the decisions of whether the individual needs screening and when he/she should initiate screening (for example, with EBV serology test).

To expand the catalog of NPC genetic variants to be used in NPC risk prediction, we initiated the Chinese Nasopharyngeal Carcinoma Collaboration study and performed the largest, to-date, genome-wide association study (GWAS) on NPC. We aimed to identify and replicate novel genetic variants in independent populations for constructing a robust PRS. Furthermore, we evaluated the performance and utility of the newly developed PRS for NPC risk stratification in endemic and non-endemic areas and explored the potential applications of the PRS for NPC screening in a prospective cohort from endemic regions in China.

## Results

### Genome-wide association analysis identifies novel NPC risk loci

**Fig. 1:**
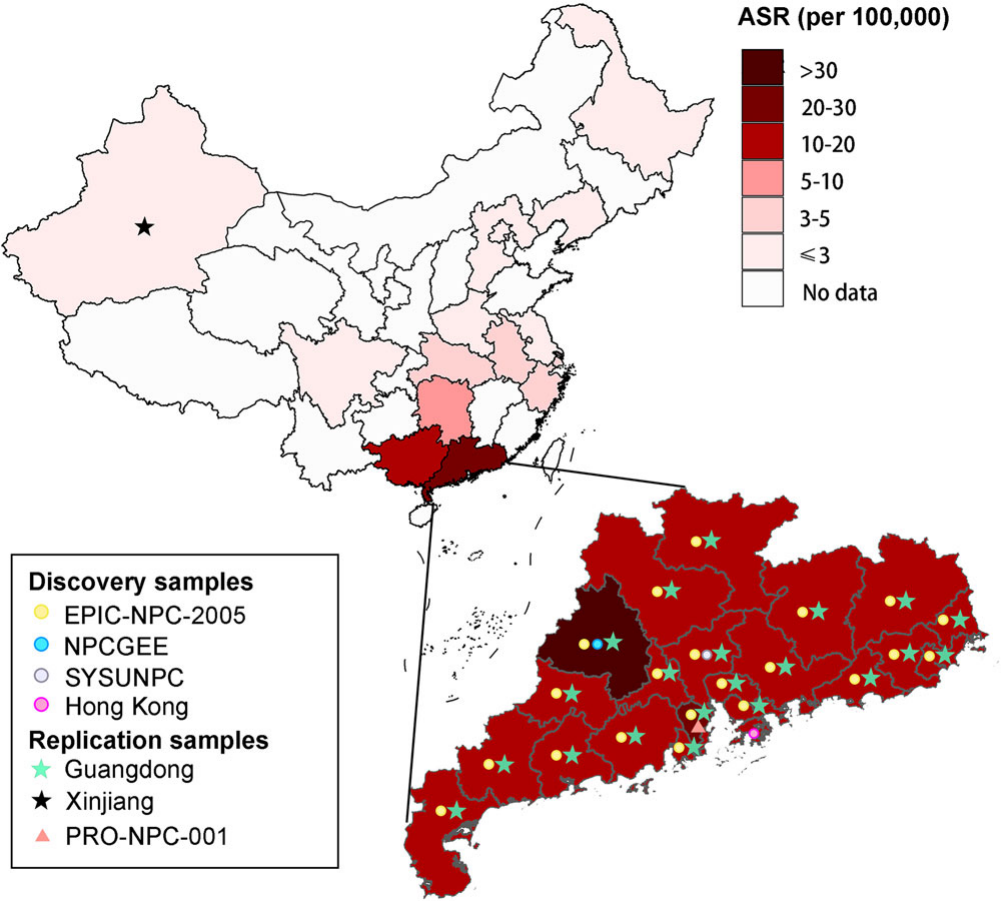
The population distribution of the study. ASR: the estimated age-standardized (world population) incidence rates of nasopharyngeal carcinoma in China. Data source: Cancer incidence in five continents Volume XI (http://ci5.iarc.fr/CI5-XI/Default.aspx).

**Fig. 2:**
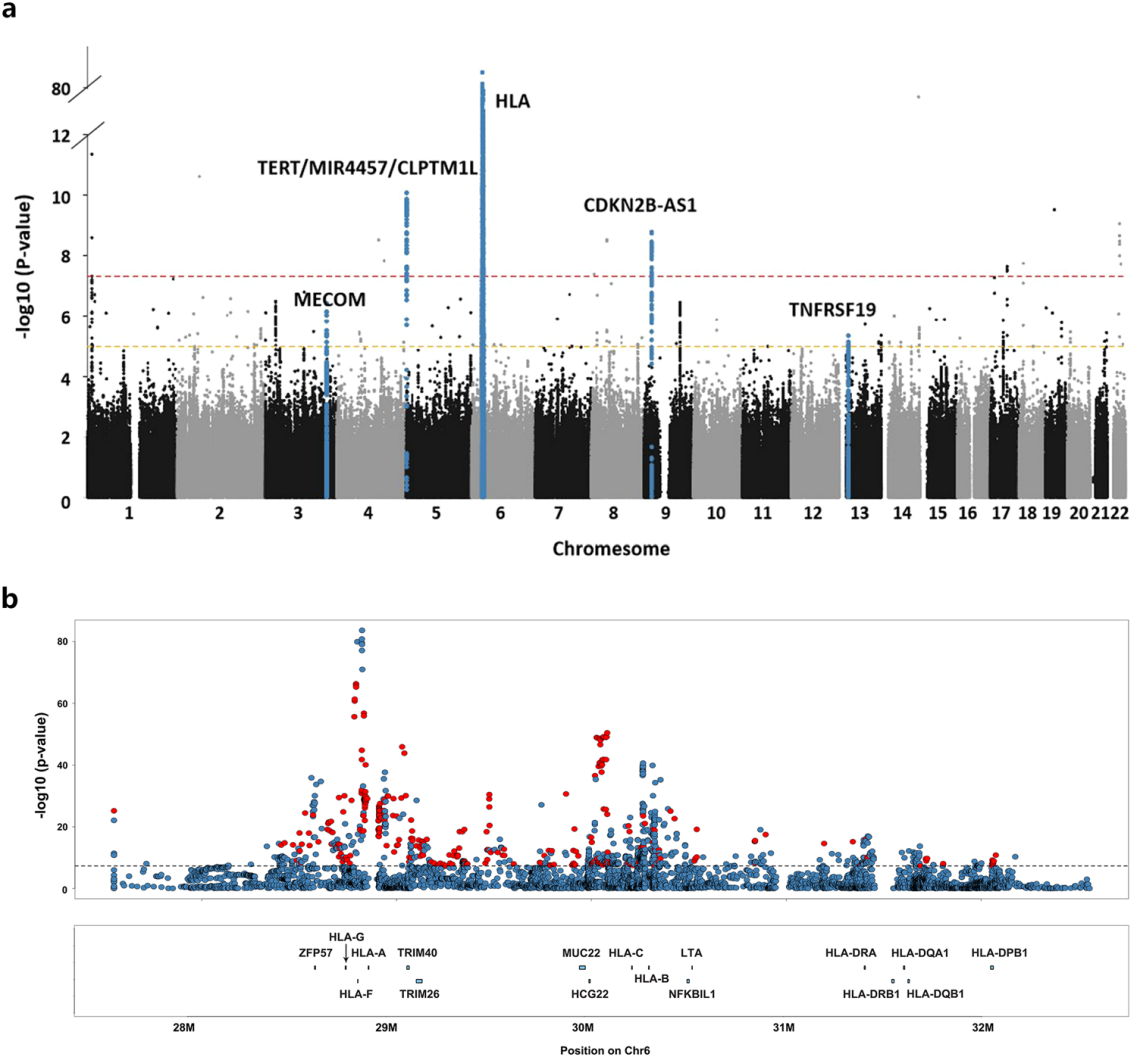
Manhattan plots showing -log10 *P* values for meta-analysis of NPC risk for (a) the whole genome and (b) the HLA region. Unconditional logistic regression analysis was conducted for each study by adjusting age, sex, and top PCs. The fixed-effect meta-analysis was performed to combine the results. All the tests were two-sided. The *P* values were shown with no adjustments for multiple comparisons. The red horizontal lines indicate genome-wide significance level (*P* = 5.0 × 10^−8^), and the yellow horizontal lines indicate genome-wide suggestive significance level (*P* = 1.0 × 10^−5^). The blue dots represent the reported susceptibility loci. The red dots in (b) mark the SNPs with *P* < 5.0 × 10^−8^ in the conditional regression analysis.

**Fig. 3:**
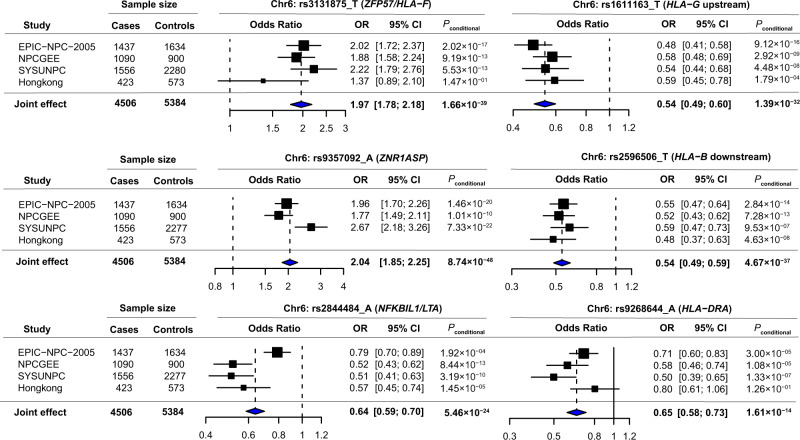
Novel variants associated with NPC risk in the meta-analysis of GWAS. Stepwise conditional meta-analysis was used to calculate the OR for each SNP. All the tests were two-sided. The *P* values were shown with no adjustment for multiple comparisons. OR odds ratio. For the Hong Kong sample, rs9357092 was excluded due to its low imputation info score.

Genome-wide meta-analysis of four population samples (Fig. 1) including 4506 NPC patients and 5384 controls identified 1400 associations SNPs surpassing the GWAS threshold (*P* < 5.0 × 10^−8^) (Fig. 2). The previously identified risk loci were also well replicated (3q26, 5p15, 6p21.3, 6p22.1, 9p21, 13q12, shown in Supplementary Table 1). Stepwise conditional meta-analysis revealed nine HLA SNPs surpassing *P*_*joint*_ < 5.0 × 10^−8^. When conditioned on the previously reported HLA SNPs, six of these SNPs have additional contribution to NPC risk (Fig. 3; Supplementary Table 2; Supplementary Fig. 1), including rs3131875 (*ZFP57/HLA-F*: OR = 1.97, 95% CI = 1.78–2.18, *P*_*conditional*_ = 1.66 × 10^−39^), rs1611163 (upstream of *HLA-G*: OR = 0.54, 95% CI = 0.49–0.60, *P*_*conditional*_ = 1.39 × 10^−32^), rs9357092 (*ZNR1ASP*: OR = 2.04, 95% CI = 1.85–2.25, *P*_*conditional*_ = 8.74 × 10^−48^), rs2596506 (*HLA-B* downstream: OR = 0.54, 95% CI = 0.49–0.59, *P*_*conditional*_ = 4.67 × 10^−37^), rs2844484 (*NFKBIL1/LTA*: OR = 0.64, 95% CI = 0.59–0.70, *P*_*conditional*_ = 5.46 × 10^−24^) and rs9268644 (*HLA-DRA*: OR = 0.65, 95% CI = 0.58–0.73, *P*_*conditional*_ = 1.61 × 10^−14^). We consider these six HLA SNPs to be novel SNPs with additional contribution to NPC risk.

### GWAS-derived PRS enables the effective identification of NPC high-risk individuals

**Fig. 4:**
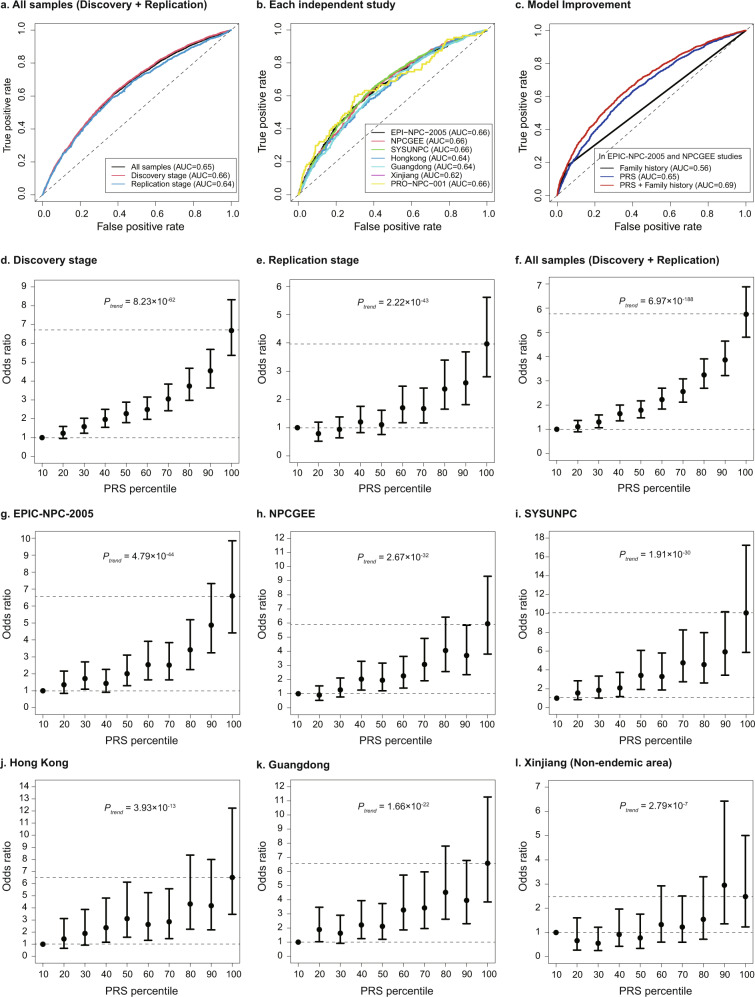
GWAS-derived polygenic risk score enables effective identification of the high-risk individuals and predicts NPC risk with moderate accuracy. **a** AUCs of the PRS in the combined samples from the discovery and replication stages; (**b**) AUCs of the PRS in each independent population; (**c**) AUCs of different models showed that the PRS provided additional predictive ability beyond the risk factor of self-reported NPC family history; (**d**–**f**) ORs of developing NPC for each PRS decile in the samples from discovery stage (*n* = 9890, Fig. **d**), replication stage (*n* = 2893, Fig. **e**) and combined stage (*n* = 12,783, Fig. **f**); (**g**–**l**) ORs of developing NPC for each PRS decile in each independent sample of EPIC-NPC-2005 (*n* = 3071, Fig. **g**), NPCGEE (*n* = 1990, Fig. **h**), SYSUNPC (*n* = 3833, Fig. **i**), Hong Kong (*n* = 996, Fig. **j**), Guangdong (*n* = 2192, Fig. **k**) and Xinjiang (non-endemic area) (*n* = 701, Fig. **l**). Multiple logistic regression analysis was used to calculate the ORs adjusted for sex and age. All the tests were two-sided. The solid dots in the center for the error bars are the OR values, and the error bars are the corresponding 95% confidence intervals of the ORs. The dashed lines represent the OR values for samples with PRS ≥ 90% (upper line) and PRS < 10% (lower line). PRS polygenic risk score. Source data of (**d**–**l**) are provided in the Source Data file.

The six newly identified and six previously identified SNPs were incorporated into the PRS model (Supplementary Table 3). The area under the curve (AUC) of the PRS was 0.65 (95% CI = 0.64–0.66) in the combined samples of the discovery stage and ranged from 0.64 to 0.66 in each of the studies (Fig. 4a, b). The PRS was well-replicated in two another independent case-control samples from NPC endemic (Guangdong sample: AUC = 0.64) and non-endemic areas (Xinjiang sample: AUC = 0.62), as well as in the prospective NPC screening cohort (PRO-NPC-001: AUC = 0.66) (Fig. 4b). By adding the PRS to the model including NPC family history only, the AUC of the model substantially increased from 0.56 to 0.69. The increment of the expected information for discrimination (*Λ*) is 0.2 bits (*P* < 0.005), showing that the PRS significantly improved the prediction of NPC risk (Fig. 4c, Supplementary Fig. 2 and Supplementary Table 4).

In the combined samples of the discovery stage, participants in the top 10% of the PRS had a 6.68-fold NPC risk (95% CI = 5.37–8.32) compared with those in the bottom 10% (Fig. 4d). A similar dose-response relationship of the PRS was also observed in the replication samples (Fig. 4e). In the combined samples from both discovery and replication stages, participants in the top 10% of the PRS had a 5.75-fold NPC risk (95% CI = 4.80–6.88) compared with those in the bottom 10% (Fig. 4f). The participants in the top 10% PRS had 495–905% excess NPC risk compared to those in the bottom 10% in endemic areas. This pattern was robust and consistent in each of the six separate samples from both endemic and non-endemic regions (*P*_*trend*_ ranging from 2.79 × 10^−7^ to 4.79 × 10^−44^) (Fig. 4g–l). When we further evaluated the PRS in the prospective NPC screening cohort, participants in the top 10% PRS had an HR of 9.17 (95% CI = 3.19–26.35, *P* = 3.89 × 10^−5^) compared with those in the bottom 20% PRS (Supplementary Table 5).

### Utility of PRS in NPC screening

**Fig. 5:**
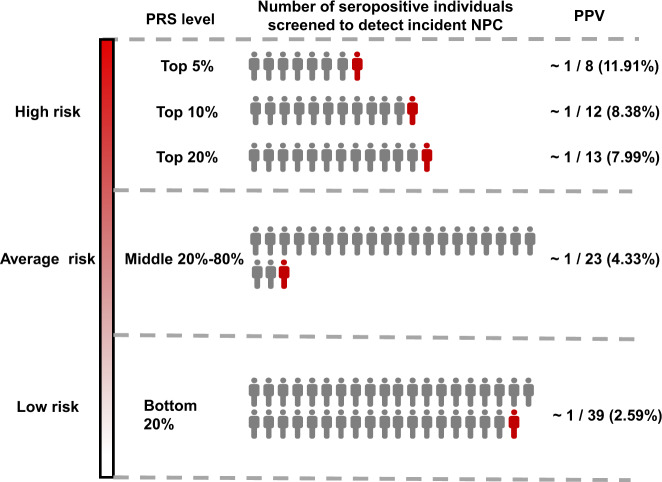
Impact of polygenic risk score on positive prediction value of EBV serological test for NPC. The numbers of seropositive individuals screened (colored gray and red) relative to the numbers of individuals receiving a benefit from the more thorough clinical assessments (colored red) are shown by the PRS subgroups (top 5th percentile, top decile, top quintile, middle three quintiles and bottom quintile of polygenic risk score). PRS polygenic risk score; PPV predictive prediction value.

In the PRO-NPC-001 screening cohort, an average PPV of 4.84% was found based on 70 incident NPC cases among 1445 participants with high risk indicated by EBV serology, and the negative predictive value (NPV) for EBV test was 99.9% (27,638 were true controls among the 27,657 EBV negative test). By incorporating the PRS into EBV-serology-based screening, the PPV was 2.59% for seropositive participants in the lowest 20% of PRS (39 seropositive individuals screened to detect one incident NPC). However, the PPV was 7.99% for seropositive subjects in the top 20% of the PRS (13 seropositive individuals screened to detect one incident NPC), 8.38% for the top 10% of the PRS (12 seropositive individuals screened to detect one incident NPC), and 11.91% for the top 5% of the PRS (8 seropositive individuals screened to detect one incident NPC), all of which were higher than the average value of 4.84% (Fig. 5). Among the remaining 27 657 participants who were defined as low risk by EBV serology, 19 subjects were missed diagnosis by EBV tests and developed to NPC during the follow-up. We found that 8 out of 19 cases (42.11%) were in the top 10% PRS, and 18 out of 19 cases (94.7%) were in the top 50% PRS (Supplementary Table 6). The AUC of the PRS in the cohort with these 19 EBV seronegative cases and 1118 randomly selected non-cancer controls reached to 0.82, although the sample size for NPC cases was relatively small (Supplementary Fig. 3).

**Fig. 6:**
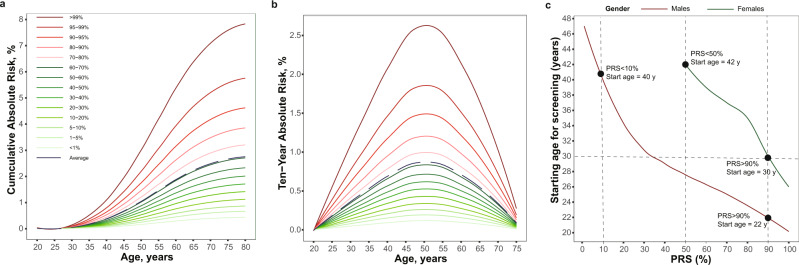
The absolute risk of developing NPC and the recommended screening initiation age based on the PRS. **a** The cumulative risk of developing NPC (*y* axis) is evaluated as an absolute risk between age 20 years and a specific age (*x* axis) for the males; (**b**) The 10-year risk is evaluated as an absolute NPC risk over the next 10 years at a particular age (shown on the *x* axis) for the males; (**c**) The recommended age to start NPC screening based on the PRS. The risk threshold to determine the age for the first screening is set to be 0.20%, the average of 10-year NPC risk for a 30-year-old subject. The red solid line is for men and the green solid line is for women. The horizontal line represents the recommended age (30 years) for the first EBV antibody test for a person with an average risk under the current screening guidelines for NPC. The three vertical lines correspond to the 10%, 50%, and 90% of the polygenic risk score in the populations. PRS, polygenic risk score. Source data of (**a**–**c**) are provided in the Source Data file.

The average cumulative risk of developing NPC during one’s lifetime (between ages 20 and 80 years) was 2.74% for males and 0.83% for females, while for subjects in the lowest and highest 1% of PRS, the corresponding cumulative risks were 0.43% and 7.79% for males, and 0.11% and 2.19% for females, respectively, making an 18-fold risk difference between the extreme PRS subgroups (Fig. 6a and Supplementary Fig. 4a). For a 30-year-old subject, the average 10-year risk of NPC was 0.20% (0.30% for males and 0.09% for females). However, for 30-year-old subjects in the lowest and highest 1% of PRS, the corresponding absolute 10-year risks were 0.05% and 0.94% for males, and 0.01% and 0.26% for females, respectively, showing an ~18-fold difference between the two extremes (Fig. 6b and Supplementary Fig. 4b).

By setting the risk threshold as the average of the 10-year NPC risk for a 30-year-old subject (0.20%), we estimated the recommended starting age of first screening given the PRS. The recommended starting age for males was 22 years for those in the top 10% PRS subgroup and 40 years for those in the bottom 10% PRS subgroup (Fig. 6c). The corresponding age for women was 30 years in the top 10% PRS subgroup, while females in the bottom 50% PRS subgroup did not reach the risk threshold in their entire lifetime (Fig. 6c).

## Discussion

In this study, we newly developed and replicated a PRS to predict an individual’s inherent genetic risk for developing NPC with relatively good performance and firstly evaluated the utility of the PRS in NPC screening from one prospective cohort. The PRS is powerful to identify high-risk individuals and decrease the missed diagnostic rate of the EBV-based screening tests, while avoiding unnecessary screening and therefore improving screening efficiency. The PRS represents a personalized genetic assessment, which should be calculated once in the lifetime, long before the onset of NPC, and thus could inform the clinical decisions of whether and when to initiate screening for a given individual.

The PRS could identify individuals with relatively high risk and the PRS-informed individualized screening could be used to identify who would benefit from EBV-serology-based screening. Participants from endemic areas in the top 10% of PRS compared with those in the bottom 10% had a 5.95- to 10.05-fold risk for developing NPC. In contrast, the risk gradient was 2.48-fold in a non-endemic area, suggesting that our PRS also possesses the promising ability for NPC risk prediction in non-endemic areas. Our novel PRS had relatively good performance (AUC = 0.66) compared with the PRS derived for colorectal cancer (AUC = 0.55–0.60)^27,28^, breast cancer (AUC = 0.53–0.69)^29–33^, prostate cancer (AUC = 0.57–0.67)^30,34^, lung cancer (AUC = 0.55)^35^ and esophageal adenocarcinoma (AUC = 0.60)^36^.

Additionally, by incorporating the PRS into EBV-serology-based screening, the PRS could stratify the seropositive individuals into different risk subgroups and identify the individuals who would benefit from more thorough clinical assessments, such as nasopharyngeal fiberscope and even a pathological biopsy. The PPV of tumor biomarkers for cancer screening programs was relatively low given the low incidence of cancer. For instance, the PPV of a stool DNA test for colorectal cancer screening was 3.70%^37^, and that using fetoprotein test for hepatocellular carcinoma screening was 1.66%^38^. In this study, the PRS stratification could substantially improve the PPV of the existing screening strategies for NPC. The PPV of the EBV antibody test alone in our cohort was 4.84%, but it increased up to 11.91% for participants in the highest 5% of PRS.

The PRS is an indicator of the utility of screening and could be informative in deciding when to participate in NPC screening for a given individual. The proposed PRS model would be instrumental in improving informed precision decisions on NPC screening. Indeed, our results provided strong evidence to recommend males in the top 10% of genetic risk based on the PRS to start NPC screening at the age of 22 years, because their 10-year risk exceeds the threshold derived from the current guidelines implemented in China. The PRS using genetic information might offer new possibilities for the precision management of complex diseases. The increased genetic risk for diseases could be discovered at younger ages, much earlier before clinical risk factors become manifest, thereby providing a potent instrument for primary and secondary prevention for those high-risk individuals. Strong evidence also suggested that inherited risk could be successfully modulated by a healthy lifestyle (6, 7) or medication use (8, 9). In this study, the cost for one NPC PRS test is similar to that of one EBV test in Mainland China, for example, US$7.7 for each PRS test (Supplementary Note). However, the EBV test should be repeated over time, and the PRS test only needs to be performed once in a lifetime. So, we infer that a combination of the EBV test and the PRS test may be a cost-effective and feasible NPC screening strategy.

We identified nine conditionally significant SNPs in the HLA region, indicating a potential biological role of some novel non-classical HLA genes. For example, LTA (implicated by rs2844484), also called tumor necrosis factor-beta (TNF β), is a cytokine that mediates a large variety of inflammatory, immunostimulatory, and antiviral responses, and also plays a role in the regulation of cell survival, proliferation, differentiation, and apoptosis. TRIM40 (implicated by rs9261506), a negative regulator against inflammation, plays a role in carcinogenesis of the gastrointestinal tract and was also reported to enhance viral replication through inhibition of innate antiviral immune responses^39^. Taking these lines of evidence together, our novel findings suggest that susceptibility genes for NPC development and EBV infection may act in concert.

Our study had several limitations. First, the PRS here was applied in combination with the EBV antibody test alone. In a recent prospective study, plasma EBV DNA test was found to be useful for NPC screening with a relatively high PPV compared with EBV antibody test^40^. Since the PRS could predict an individual’s inherent genetic risk for developing NPC and therefore influencing pretest probability, we think it could also be expected to add value to other screening strategies, such as plasma EBV DNA copies, nasopharyngeal EBV DNA copies, EBV microRNAs, or some additional screening test independent of EBV test. Further study is needed to evaluate the value of combining the PRS with different screening strategies (for example, plasma EBV DNA test). Second, our current predictive model by the case-control study includes only the family history of NPC, but no other identified risk factors. A well-designed cohort study including more established risk factors, such as age, sex, smoking, and diet, might further improve the current model. Moreover, the number of NPC patients was still relatively small in our prospective cohort, which would result in diminished statistic power and should be prudent to present the range of the PRS. An extended cohort with a larger sample size and longer follow-up would be better to evaluate the validity of the PRS model in NPC risk stratification and screening. Last, we evaluated risk loci identified only in Chinese populations, albeit from NPC endemic and non-endemic regions. International cooperation is warranted to explore the genetic variants and the biological mechanisms through which they affect NPC risk in multiple ethnic populations worldwide. Overall, although we provided evidence of a potential application of the PRS in NPC screening, it’s just an initial study, and much work remains in establishing its discriminative ability in the general population. In the future, a more precise PRS model, especially a comprehensive model integrated with individual risk exposures and EBV biomarkers, should be developed and thoroughly evaluated. Most importantly, rigorous clinical trials are warranted to assess its clinical applications strictly.

In conclusion, we developed and replicated a GWAS-derived PRS for personalized genetic assessment of NPC risk. The PRS could identify high-risk individuals who would benefit from screening and inform clinical decisions of whether and when to participate in NPC screening for a given individual. The PRS might therefore pave the way for personalized risk prediction prevention, screening, and counseling. These findings may further benefit the deep understanding of the etiology for nasopharyngeal carcinoma and act as a potential application example in other EBV-associated diseases, especially for future individualized screening.

## Methods

The Institutional Review Board of Sun Yat-Sen University Cancer Center approved this study. Informed consent was obtained from all study participants.

### Study design and participants

The Chinese Nasopharyngeal Carcinoma Collaboration study (ChiCTR1900027868) includes 6059 incident NPC cases and 7582 hospital- and population-based non-NPC controls from regions with different NPC incidence rates in China. For the PRS construction, 4 GWAS populations were included from regions in southern China with the highest NPC incidence, including the EPI-NPC-2005 sample (1614 cases and 1819 controls)^41,42^, NPCGEE sample (1098 cases and 991 controls)^43^, SYSUNPC sample (1617 cases and 2610 controls)^44,45^ and Hong Kong sample (426 cases and 573 controls)^46,47^. For the PRS replication, another two independent samples were included, one from NPC endemic area (Guangdong sample: 954 cases and 1238 controls) and the other from NPC non-endemic area (Xinjiang sample: 350 cases and 351 controls). The participants’ geographical distribution and demographic characteristics are shown in Fig. 1 and Supplementary Table 7. According to the World Health Organization classification criteria for NPC, all cases were histologically confirmed by at least two pathologists. The controls in the study populations were self-reported cancer-free individuals who were frequency matched to cases by geographical region and ancestry. Recruitment and study methods for each study are shown in the Supplementary Note.

To evaluate the potential application of the PRS in NPC screening, we used a prospective cohort (PRO-NPC-001) that has recruited individuals from NPC endemic areas in southern China since 2009^12,17^. Detailed demographic characteristics of the participants are shown in Supplementary Table 8. In brief, 29,413 participants were included and screened with tests of two anti-EBV antibodies (VCA-IgA and EBNA1-IgA). With a median follow-up time of 7.33 years (IQR 3.20–7.87), 1756 (5.97%) participants were identified as high-risk individuals by EBV tests and were referred for further clinical examination. Then, 70 participants were histologically confirmed as NPC. Among the remaining 27,657 participants identified as low-risk individuals, 19 were missed diagnosis by EBV tests and eventually confirmed as NPC patients during the follow-up. All the 89 incident cases and 1118 randomly selected controls, frequency matched to cases by sex and age, were used to calculate the discriminatory power of the PRS in this screening cohort. In addition, to evaluate the discriminatory power of the PRS, especially for those missed diagnosed individuals by EBV tests, all the 19 EBV seronegative cases and the same control group were used for the PRS analysis.

### Genotyping

We used multiple genotyping arrays (Illumina Infinium Global Screening Array, Human610-Quad BeadChip, and Infinium Asian Screening Array) for genome-wide genotyping in the four study samples (Supplementary Table 7). We conducted standard quality control at subject and SNP levels (Supplementary Notes). In brief, low-quality variants were removed, and subjects were excluded for the following reasons: (1) unintended technical errors or low genotyping quality; (2) estimated to be biologically related to other subjects and with lower call rates; (3) ancestral structure deviated from that of the underlying study population (Supplementary Fig. 5).

To improve the density of genotypes and maximize the number of overlapping SNPs among samples genotyped by different arrays, we conducted imputation for each dataset with the same array. We applied different imputation methods for non-MHC and MHC regions (29–34 Mb on chromosome 6 according to *Homo sapiens* genome assembly GRCh37). For non-MHC regions, we applied SHAPEIT (v2.12) for phasing and IMPUTE2 for imputation using the 1000 Genome Phase III integrated variant set of the entire population as a reference panel. For the MHC region, we applied SNP2HLA for imputation, using the Han Chinese reference panel which includes data from 10,689 healthy individuals provided by the Beijing Genomics Institute (BGI). We further excluded variants with low imputation quality or abnormal allele frequencies (Supplementary Table 9). For non-HLA SNPs, we used the best-guess genotypes (maximum posterior probabilities exceeding a threshold of 0.9) and applied plink to analyze these data in further analysis. For HLA SNPs, we used the dosage data and applied R software using a logistic regression model in the analysis.

After strict quality control and imputation, 4506 cases and 5384 controls with the corresponding numbers of SNPs in each study sample were included for further analysis (Supplementary Table 10). The SNPs were directly genotyped using the iPLEX Sequenom MassARRAY platform for the PRS model application. We used Sanger sequencing for cross-validation of the genotyping among different platforms (Supplementary Table 11).

### Polygenic risk score

A PRS for NPC was derived by integrating previously known^19–26^ and newly discovered genome-wide significant SNPs. A total of 12 independent variants were included in the PRS calculation based on the GWAS results (EPIC-NPC-2005, NPCGEE, SYSUNPC, and Hong Kong samples). The PRS was then replicated in independent case-control samples from two areas with distinct rates of NPC incidence (Guangdong and Xinjiang samples) and the prospective screening cohort (PRO-NPC-001). The detailed process for the PRS construction is illustrated in Supplementary Fig. 6 and Supplementary Notes. The PRS was generated by multiplying the genotype dosage of each variant risk allele with its respective weight (the log odds ratio of each risk allele), and summing the results of all variants^45^.

### The absolute risk of NPC incidence and starting age for the first screening

We modeled the absolute risk of NPC in high-risk areas of southern China by combining the estimates of OR parameters obtained from our GWAS studies. The risk allele frequencies and ORs for the included variants are shown in Supplementary Table 3. The age-specific NPC incidence rates for males and females were derived from the International Agency for Research on Cancer (IARC)’s Cancer Incidence in Five Continents, Volume XI (http://ci5.iarc.fr/CI5-XI/Pages/age-specific-curves_sel.aspx, shown in Supplementary Data 1). We projected the distribution of absolute age-specific cumulative NPC risks at different percentiles of the PRS^48–50^. The current recommended starting age for NPC screening by the Chinese Ministry of Health was 30 years old. By setting a risk threshold as the average of the 10-year NPC risk for a 30-years old man (0.30%) and woman (0.09%), that is, (0.30% + 0.09%) / 2 = 0.20%, we estimated the recommended starting age of first screening given the PRS.

### Statistical analysis

For the discovery samples, the per-allele ORs and standard errors (SEs) were calculated using logistic regression with PLINK software or R software based on the additive assumption in the discovery stage. Fixed-effect meta-analysis was performed to estimate the combined effect of the variants. We used stepwise conditional meta-analysis to identify independent SNPs. The genome-wide significance threshold was set at *P* < 5.0 × 10^–8^. Categories of the PRS were designated by centile from the controls of the discovery stage and all centiles refer to these samples. For the replication samples, ORs and 95% CIs of NPC risk for the PRS subgroups were calculated by logistic regression with adjustment for sex and age. To test the association between the PRS and incident NPC in the PRO-NPC-001 cohort, we calculated hazard ratios (HRs) and 95% CIs adjusting for sex and age. Participants were classified into 10 deciles according to the distribution of the PRS, and those with the lowest PRS were used as the reference group. Due to the limited number of incident NPC cases in the PRO-NPC-001 cohort, we used the bottom 20% of the PRS as the reference group to increase statistical power.

To compare the performance of a model including self-reported family history of NPC only and a model incorporating NPC family history and the PRS, we calculated the expected information for discrimination (expected weight of evidence, denoted as *Λ*)^51^ in the available studies of EPIC-NPC-2005 and NPCGEE, considering that the contributions of independent variables to predictive performance are additive on the scale of *Λ*. To explore the utility of the PRS in NPC screening, 1445 out of all the 1756 high-risk individuals with available biospecimens in PRO-NPC-001 were used for the further PRS analysis and positive predictive value (PPV) calculation.

All analyses were conducted using R software (3.6.1). Two-sided *P* values were reported for all statistical analyses. Additional detailed calculation procedures are presented in Supplementary Notes.

### Reporting summary

Further information on research design is available in the Nature Research Reporting Summary linked to this article.

## Supplementary information

Supplementary Information

Description of Additional Supplementary Files

Supplementary Data 1

Reporting Summary

## Data Availability

The baseline patient information and genetic information data have been deposited in the Research Data Deposit public platform (www.researchdata.org.cn, accession number: RDDA2020001599). The raw genotype and phenotype data has been uploaded to The European Genome-phenome Archive (EGA) dataset (EGAS00001006062; EGAS00001006102). The summary statistics that support the findings of this study have been deposited in the NHGRI-EBI GWAS Catalog dataset [https://www.ebi.ac.uk/gwas/, accession number: GCST90093313]. Source data are provided with this paper.

## Source data

Source Data
