# Supplementary material for: A polygenic risk score for nasopharyngeal carcinoma shows potential for risk stratification and personalized screening
Source: Nat Commun. 2022 Apr 12;13:1966. doi: 10.1038/s41467-022-29570-4 (PMC9005522; doi:10.1038/s41467-022-29570-4)
Supplement: Supplementary file 1 — Supplementary Information [file 41467_2022_29570_MOESM1_ESM.pdf]

Supplementary Material

Supplementary Method 1. Study populations

Supplementary Method 2. Methods for PRS construction and replication

Supplementary Figure 1. The linkage disequilibrium among the 9 identified HLA SNPs in the stepwise conditional meta-analysis and the 16 reported HLA SNPs

Supplementary Figure 2. The PRS provided additional predictive ability beyond the model using traditional risk factors (self-reported family history of NPC)

Supplementary Figure 3. The AUC of PRS for NPC cases who were defined as low-risk and randomly selected non-cancer controls by EBV tests in PRO-NPC-001 cohort

Supplementary Figure 4. The estimated cumulative risks and 10-year risks of developing nasopharyngeal carcinoma by PRS in the females

Supplementary Figure 5. Principal components (PC) analysis of the GWAS samples

Supplementary Figure 6. Flowchart for the study design

Supplementary Table 1. Nasopharyngeal carcinoma risk signals previously reported at genome-wide significance

Supplementary Table 2. The stepwise conditional analysis found 9 SNPs associated with NPC risk in HLA region, and 6 of them (marked by *) remained statistical significance after adjusting the previously reported HLA SNPs

Supplementary Table 3. The odds ratio and the risk allele frequencies of the SNPs in the model for cumulative NPC incidence risk estimation

Supplementary Table 4. Incremental contribution of polygenic score to NPC detection compared with family history in the EPI-NPC-2005 samples, the NPCGEE samples, and the combined samples

Supplementary Table 5. The hazard ratios between PRS and NPC risk in PRO-NPC-001 cohort

Supplementary Table 6. The number and proportion of missed diagnosis NPC cases who were defined as low-risk by EBV tests in PRO-NPC-001 cohort in different PRS subgroup

Supplementary Table 7. Demographic information and genotyping information for the six samples

Supplementary Table 8. Demographic characteristics of 29 413 participants in PRO-NPC-001 cohort

Supplementary Table 9. The imputation quality score for the six novel SNPs in the 4 GWAS studies

Supplementary Table 10. Summary statistics of the samples and imputation SNPs in the discovery stage

Supplemental Table 11. The Consistency of SNP genotypes between GWAS genotyping and sanger sequencing.

Supplementary Method 1. Study populations

1.1 Study populations in the PRS construction stage

1.1.1 EPI-NPC-2005 sample

EPI-NPC-2005 study was a multicenter case-control study recruiting subjects from 21 regions in Guangdong province between January 2005 and December 2007, aiming to systematically investigate the risk factors of NPC. The details of the study design and methods had been described previously [1, 2]. We had performed a GWAS involving 1 615 cases and 1 025 controls of 21RCCP in 2010 [3]. In the current study, we newly genotyped the remaining 825 controls by GSA array to increase statistical power. Briefly, all these NPC cases were histologically confirmed by at least two pathologists according to the World Health Organization (WHO) classification. Cancer-free controls were recruited from those participating in the community physical examination screening of non-communicable diseases from local physical examination centers and were frequency-matched to cases according to age, gender, and geographic regions. In total, 1 614 cases and 1 819 controls with blood samples were used for DNA extracting and genotyping in the discovery stage.

1.1.2 NPCGEE sample

The Nasopharyngeal carcinoma Genes, Environment, and EBV (NPCGEE) study is a collaborative population-based case-control study conducted in Zhaoqing and Wuzhou areas from March 2010 to November 2014. A detailed description of this project has been published previously [4]. Newly diagnosed and histopathologically confirmed NPC patients were identified through a rapid case ascertainment system during the study period. Using current population-based registries, the controls were randomly selected every 6-12 months from the study base that gave rise to the cases, frequency-matched by age (in 5-year groups), sex, and residential area. 1 098 cases and 991 controls from NPCGEE in the Zhaoqing area with saliva samples were used for DNA extraction and genotyping by GSA array in the discovery stage.

1.1.3 SYSUNPC sample

The Sun Yat-sen University Cancer Center Nasopharyngeal carcinoma (SYSUNPC) case-control population was a hospital-based case-control study. 1 661 non-metastatic NPC patients were recruited between April 2009 and December 2015 from an NPC-specific database within the Big-data intelligence framework at Sun Yat-sen University Cancer Centre. Detailed information regarding the big-data platform has been previously reported [5]. The cancer-free controls were selected from those participating in the community screening of non-communicable diseases (the Guangzhou GSA project) [6]. 1 617 cases from the NPC-specific big-data platform and 2 610 controls from the Guangzhou GSA project with blood samples were used for DNA extracting and genotyping by GSA array in the discovery stage.

1.1.4 Hong Kong sample

The Hong Kong Area of Excellence nasopharyngeal carcinoma (HKAoENPC) case-control study was a multicenter case-control study. The methods of this case-control study have been described elsewhere [7, 8]. NPC cases were incident patients diagnosed with histological and/or radiological evidence in the past three months to minimize the effects of recall bias and lifestyle changes secondary to NPC between July 2014 and September 2017 in the oncology departments of the five major regional hospitals. The controls were frequency-matched non-NPC patients or referrals of a new health complaint in the past 12 months in specialist outpatient clinics, or new inpatients admitted in the past 3 months in the same hospitals, following the AsiaLymph guideline of the US National Cancer Institute. 426 cases and 573 controls with blood samples were used for DNA extracting and genotyping by ASA array in the discovery stage.

1.2 Study populations in the PRS replication stage

1.2.1 Guangdong sample

The Guangdong population was a hospital-based case-control study from Guangdong province, a high NPC incidence area from Southern China. The cases were from the same NPC-specific database as described in SYSUNPC population above. 954 non-metastatic NPC patients diagnosed

between April 2009 and December 2015 and 1 238 cancer-free controls selected from those participating in the community screening of non-communicable diseases were recruited. All individuals with blood samples were used for DNA extracting and genotyping in the replication stage.

1.2.2 Xinjiang sample

NPC patients were recruited from March 2012 to March 2019 from the Affiliated Tumor Hospital of Xinjiang Medical University, a low NPC incidence area from Northwest China. All the cases were histologically confirmed as incident NPC by at least two pathologists. The cancer-free controls were selected from those participating in health check-ups in the medical center. All the subjects were of Han nationality. 350 cases and 351 controls with blood samples were used for DNA extracting and genotyping in the replication stage.

1.3 NPC screening cohort

PRO-NPC-001 was a prospective NPC screening cohort in Guangdong province, Zhongshan City of Xiaolan Township since 2009. The methods of this screening cohort have been described elsewhere [9, 10]. All eligible residents in the screening group were invited to participate in the NPC screening by door-to-door invitation, community education, and television advertisements. Inclusion criteria include 1) being aged 30-59 years; 2) being Cantonese; 3) having no prevalent NPC; 4) having an Eastern Cooperative Oncology Group score of 0-2; 5) having a good physical or psychological condition and consciousness. Exclusion criteria include 1) having severe cardiovascular, liver, or kidney disease or 2) having prevalent NPC. Written informed consent was obtained from each participant before the study.

At the initial screening, participants in the screening group were invited to accept indirect mirror examination in the nasopharynx and/or lymphatic palpation (IMLP) and donated a 6-ml blood sample for two anti-EBV antibodies testing (EBNA1-IgA and VCA-IgA). Participants were divided into three groups according to their antibody levels (serological high-, medium-, and low-risk

groups). The serological high-risk and/or IMLP (+) participants at the initial screen were referred to nasopharyngeal endoscopy. The serological medium or low-risk participants at initial screening were invited to serological retesting annually in the following three years. Nasopharyngeal biopsies were also carried out if suspicious lesions were observed under endoscopy. NPC pathology was classified according to the World Health Organization classification, and TNM staging was determined according to the 2008 Staging System of China.

29 413 eligible subjects with 178 960 person-years in the screening cohort were followed up annually for NPC incidence, mortality, vital status, and immigration status through the Cancer Registry, Death Registry, and Population Registry of Zhongshan City. The positive screening ones were defined as participants with high serological risk at least once at the initial screening or the retest. With a median follow-up time of 7.33 years (IQR 3.20-7.78), 1 756 (5.97%) participants were identified as screening positive ones with high serological risk, and 70 participants among them were histologically confirmed as NPC patients. 1 445 high-risk individuals with available biospecimens were used for further PRS analysis and positive predictive value (PPV) calculation. Among the remaining 27 657 participants identified as low-risk individuals, 19 were missed diagnosis by EBV tests and histologically confirmed as NPC patients (non-keratinizing undifferentiated carcinoma) during the follow-up. All the 89 incident cases and 1 118 randomly selected controls, frequency matched to cases by sex and age, were used to calculate the discriminatory power of the PRS in this screening cohort. In addition, to evaluate the discriminatory power of the PRS, especially for those missed diagnosed individuals by EBV tests, all the 19 EBV seronegative cases and the same control group were used for PRS analysis.

Supplementary Method 2. Methods for PRS construction and replication

2.1 Genotyping, imputation and quality control

The peripheral blood/saliva/paraffin tissue sample was collected for genomic DNA extraction from each participant. We conducted genotyping using Illumina Infinium Global Screening Array, Illumina Human610-Quad BeadChip, and Illumina Infinium Asian Screening Array. The genotypes were called by using the Illumina iScan System according to the manufacturer's instructions. We excluded samples with gender discrepancy, call rates < 95%, or excessive heterozygosity rates (more than six standard deviations from the mean). We found cryptic familial relatedness by identity by descent (IBD) analysis using PLINK software [11] and excluded the member with PI_HAT > 0.25 from a pair of samples with a lower call rate. Population structure was evaluated by principal component analysis (PCA) using EIGENSTRAT software [12] based on the LD-pruned autosomal SNPs ($r^2<0.3$). We identified and removed population outliers in each study (> 6 standard deviations from the mean on any one of the top 10 PCs using five iterations). Population outliers were checked by comparing our samples and 504 East Asian individuals from 1000 Genomes Project Phase III. We excluded SNPs with duplicate markers, or call rate <95%, or minor allele frequencies (MAFs) < 0.01, or $P < 10^{-7}$ in controls or $P < 10^{-12}$ in cases in HWE test. We did a manual inspection for variants with poor Illumina intensity or clustering metrics, or deviation from the frequencies in the 1000 Genomes Project (the Phase III integrated variant set release, East Asian).

Different imputation methods were applied for non-MHC and MHC regions (29-34 Mb on chromosome 6 according to Homo sapiens genome assembly GRCh37). For non-MHC regions, following a two-step imputation method, we used SHAPEIT (v2.12) [13] for phasing and IMPUTE2 [14] for imputation. The haplotype information of the 1000 Genome Phase III integrated variant set of the entire population was used as a reference panel. For the MHC region, we applied the default parameters of SNP2HLA [15] to perform the imputation, using the HAN Chinese reference panel,

which included 10 689 healthy individuals provided by BGI [16]. After imputation, quality control was performed to exclude variants with poor imputation quality (INFO <0.3 for the non-MHC region and r^2 <0.5 for MHC region), MAF <0.005, call rate <95%, or deviation from Hardy-Weinberg disequilibrium ($P<1.0\times10^{-12}$ in cases and $P<1.0\times10^{-7}$ in controls). For the imputed variants in the MHC region, we also compared the imputed allele frequencies with the frequencies in HAN Chinese reference and excluded the abnormal variants. We used the imputed allelic genotypes of the non-MHC region and the imputed dosage genotypes of the MHC region for the downstream analysis. Sanger sequencing was used to cross-validate the genotyping among different platforms, and rs3094173 were excluded due to low concordance.

In the replication stage, individual genotypes were directly genotyped using the iPLEX Sequenom MassARRAY platform. Sanger sequencing was used for technical validation of 10 promising HLA SNPs (rs3131875, rs1611163, rs9357092, rs9261506, rs9261567, rs2251830, rs2596506, rs2844484, rs9268644 and rs3094173). One HLA SNP (rs3094173) failed in the technical validation and was excluded in the downstream analysis. The remaining SNPs were successfully validated, showing 99.4% consistency in 639 comparisons in the samples (Supplemental Table 11). We also sequenced the matched samples of blood and paraffin tissue from the same individuals to evaluate the sequence consistency of the 12 PRS-derived SNPs from different sample types. We found the consistency was 99.3% in 280 comparisons in the samples (data not shown).

2.2 Association, meta-analysis and stepwise conditional analysis

The genetic effects on NPC risk (ORs and 95% CIs) were calculated by logistic regression adjusting for age and sex, and additionally for population structure (the top eigenvectors in principal component analysis) in the 4 four studies (for EPIC-NPC-2005: PC1-PC2; for NPCGEE: PC1-PC6; for SYSUNPC: PC1-PC3; for Hong Kong population: PC1-PC3). Fixed-effect meta-analysis was performed to estimate the combined effect of the variants using the METAL software [17, 18]. We used a stepwise conditional meta-analysis to identify independent SNPs. At each locus attaining a significance level of $P<5.0\times10^{-8}$, we included the most robust statistical association signal in the region as an additional covariate for the logistic regression in each study. Pooled ORs and 95% CIs were calculated for the other variants in the region across four studies, using a fixed-effect model. If at least one additional variant achieved a significance level of $P<5.0\times10^{-8}$ in this step of meta-analysis, a second-round analysis would be performed, adding the most significant variant in this round as an additional covariate. This procedure was repeated until no more variant at the locus attained $P<5.0\times10^{-8}$. We then performed a joint multiple-variant association analysis to estimate the joint effects of the selected variants and excluded those not achieving $P_J<5.0\times10^{-8}$. Due to the extensive and complex LD structure of the MHC region, we performed the analysis overall 29-34 Mb region on chromosome 6. In the stepwise conditional meta-analysis, a total of nine HLA SNPs was found (one SNP that failed the Sanger validation mentioned above was excluded in the further analysis). Among the eight HLA SNPs after quality control, four SNPs surpassed $P<5.0\times10^{-8}$ in the meta-analysis.

2.3 PRS calculation

We derived a polygenic risk score (PRS) for NPC risk integrating the previously and newly found genome-wide significant SNPs in the discovery stage. To ensure the model's efficiency, several

criteria were used to select the variants: 1). Variants with minor allele frequency ≥ 0.01 were included; 2). Previously reported variants that were also replicated (P ≤ 0.0005) in our study were included;3). Variants achieving $P_{joint} < 5.0 \times 10^{-8}$ in joint multiple-variant association analysis were included; 4). Variants with $P_{conditional} < 0.05$ in the PRS model by multivariate analysis were included; 5). For certain SNPs that could not be successfully designed with MassARRAY in the replication stage, we used the proxy SNPs with high linkage disequilibrium ($R^2 > 0.9$). Finally, a total of 12 independent variants were kept for the PRS calculation (rs3131875, rs9268644, rs6475604, rs2844484, rs31489, rs2596506, rs9261506, rs2251830, rs9507124, rs9357092, rs2106123 and rs1611163). The PRS model was built based on the samples in the discovery stage (EPIC-NPC-2005, NPCGEE, SYSUNPC, and Hong Kong) and then replicated in external independent samples from different NPC incidence areas (Guangdong and Xinjiang sample). The detailed process is illustrated in Supplementary Figure S2. The PRS was generated by multiplying the genotype dosage of each variant risk allele by its respective weight (the ORs of each risk allele) and summing the results of all variants [6]. Effect sizes for all variants were derived from the association of patients with NPC in the present GWAS study, which was all flipped to risk alleles to calculate PRS. Briefly, the PRS was derived for each study subject using the formula:

$$\text{PRS} = \sum_{k=1}^{12} \beta_k X_k$$

Where β_k was the per-allele log odds ratio (OR) of SNP_k, X_k was the number of alleles of SNP_k (0, 1 or 2).

2.4. Absolute risk calculation

The cumulative absolute risks of developing NPC were projected for individuals with different combinations of risk variants by the cumulative incidence functions derived from the competing-risk regression. The average cumulative risk of developing NPC during the future ten years and one's lifetime (between ages 20 and 80 years) for individuals at different deciles of PRS were assessed. We described the distribution of absolute age-specific cumulative NPC risks for

individuals by different PRS subgroups. The code used to develop the model and project risks are available as part of the R software package Individualized Coherent Absolute Risk Estimator (iCARE) downloadable from: http://dceg.cancer.gov/tools/analysis/icare.

2.5 The costs for PRS laboratory test

Procedures	Reagents and consumables	CNY(￥)*	USD($)
Blood / Saliva collection	Evacuated Blood Collection Tubes (Improved medical:10180720)<br>Conical Centrifuge Tubes (BD:352098)	5	0.7
DNA extraction	Genomic DNA kit (TIANamp: DP304-03)	10	1.41
Genotyping for 12 SNPs	Sequenom iPLEX MassARRAY	40	5.63
Total		55	7.74

* As of Dec 2020, in China Mainland.

Supplementary Figures

Supplementary Figure 1: The linkage disequilibrium among the 9 identified HLA SNPs in the stepwise conditional meta-analysis and the 16 reported HLA SNPs. The colors indicate the R^2 values calculated from the samples in the discovery stage.

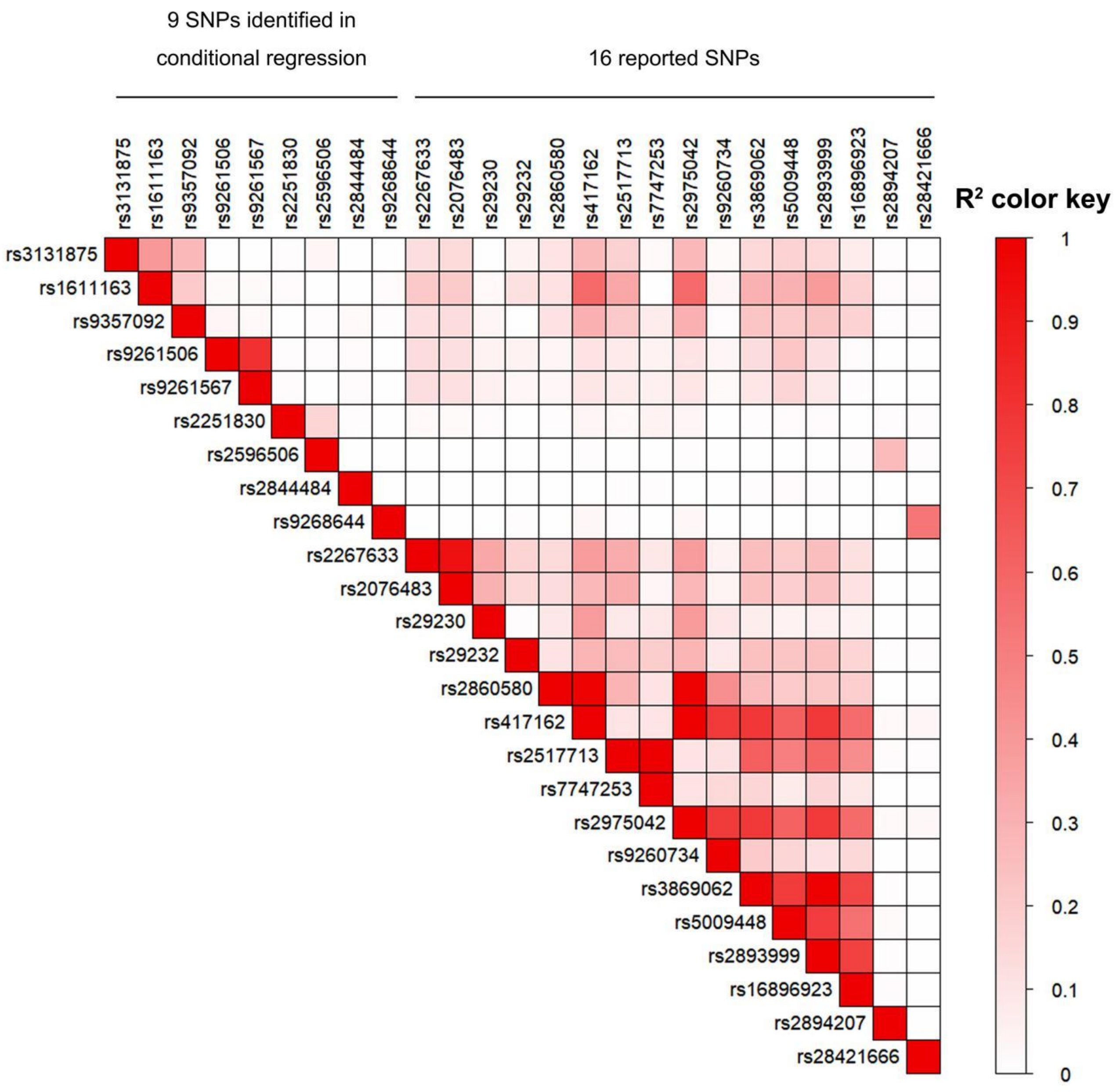

Supplementary Figure 2: The PRS provided additional predictive ability beyond the model using traditional risk factors (self-reported family history of NPC). (A) AUCs of different models in EPI-NPC-2005 sample; (B) AUCs of different models in NPCGEE sample.

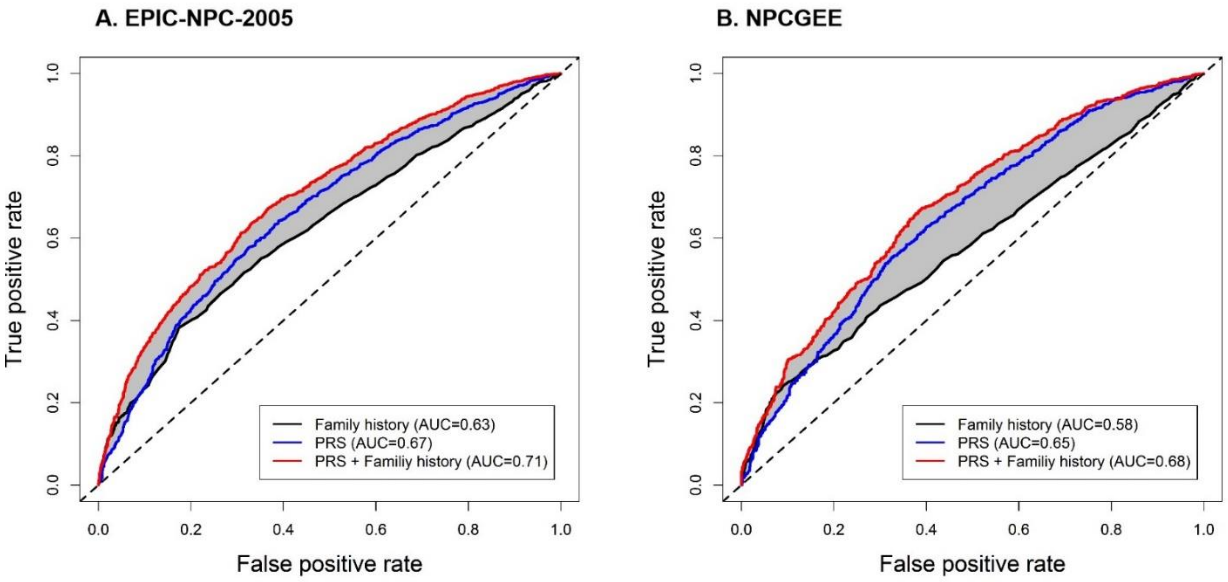

Supplementary Figure 3: The AUC of PRS for NPC cases who were defined as low-risk and randomly selected non-cancer controls by EBV tests in PRO-NPC-001 cohort.

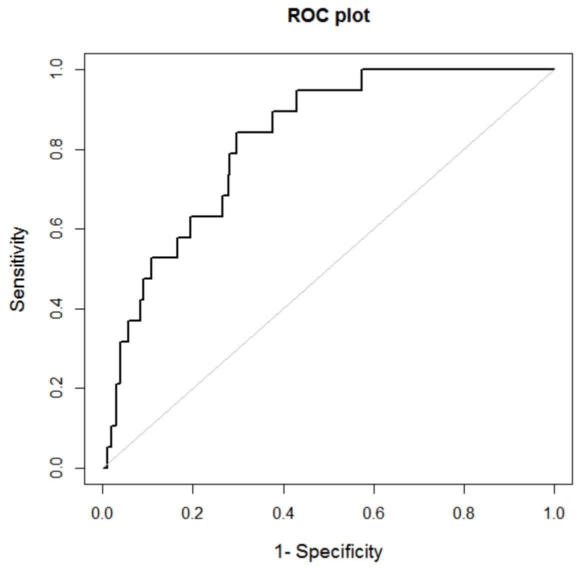

Supplementary Figure 4: The estimated cumulative risks and 10-year risks of developing nasopharyngeal carcinoma by PRS in the females. (A) The cumulative risk of developing NPC (y-axis) is evaluated as an absolute risk between age 20 and a specific age (x-axis); (B) The 10-year risk is evaluated as an absolute NPC risk over the next 10 years at a specific age (shown on the x-axis).

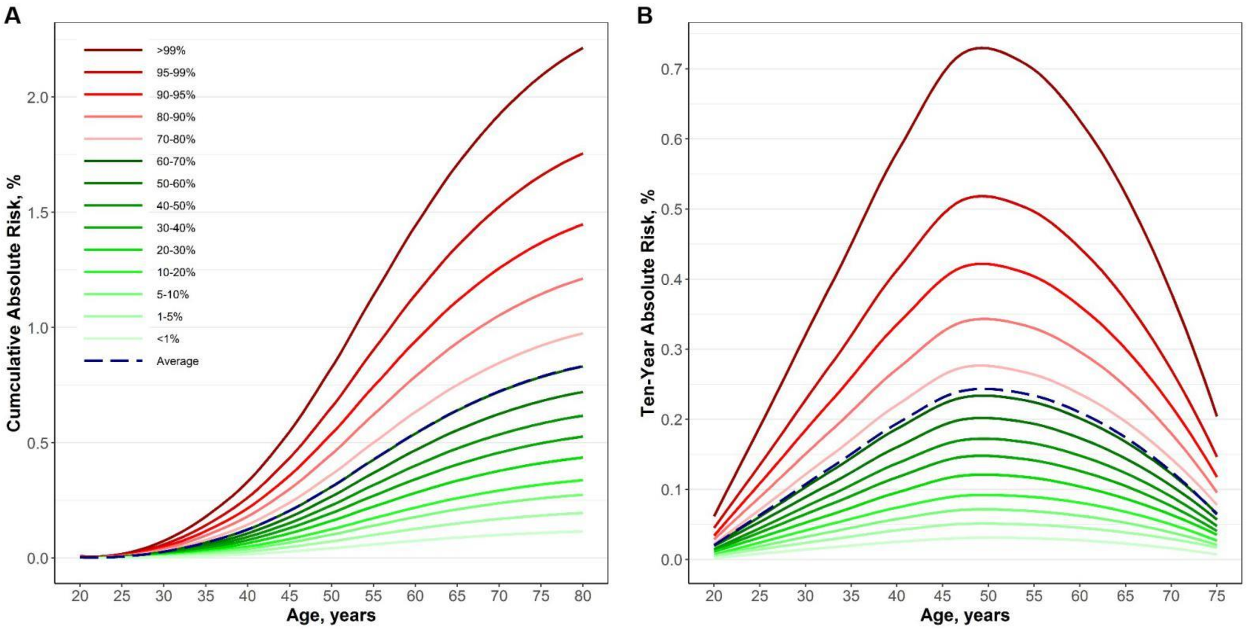

Supplementary Figure 5: Principal components (PC) analysis of the GWAS samples. Consecutive plots showed the first two PCs of the samples passed quality control from the EPI-NPC-2005 (A), or NPCGEE (B), or SYSUNPC (C), or Hongkong population (D).

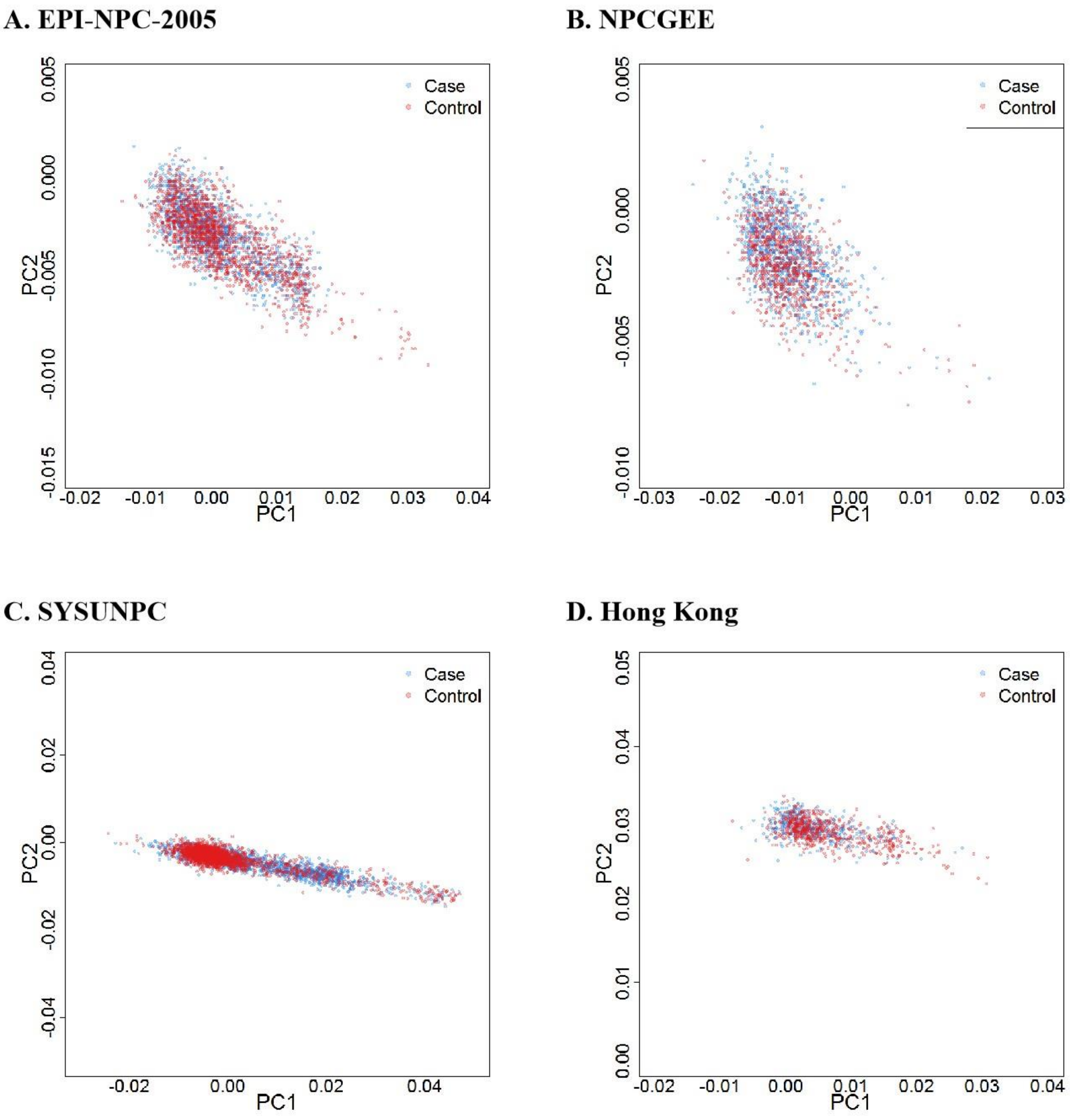

Supplementary Figure 6: Flowchart for the study design. GWAS: genome-wide association study. PRS: polygenic risk score. SNPs: single nucleotide polymorphisms.

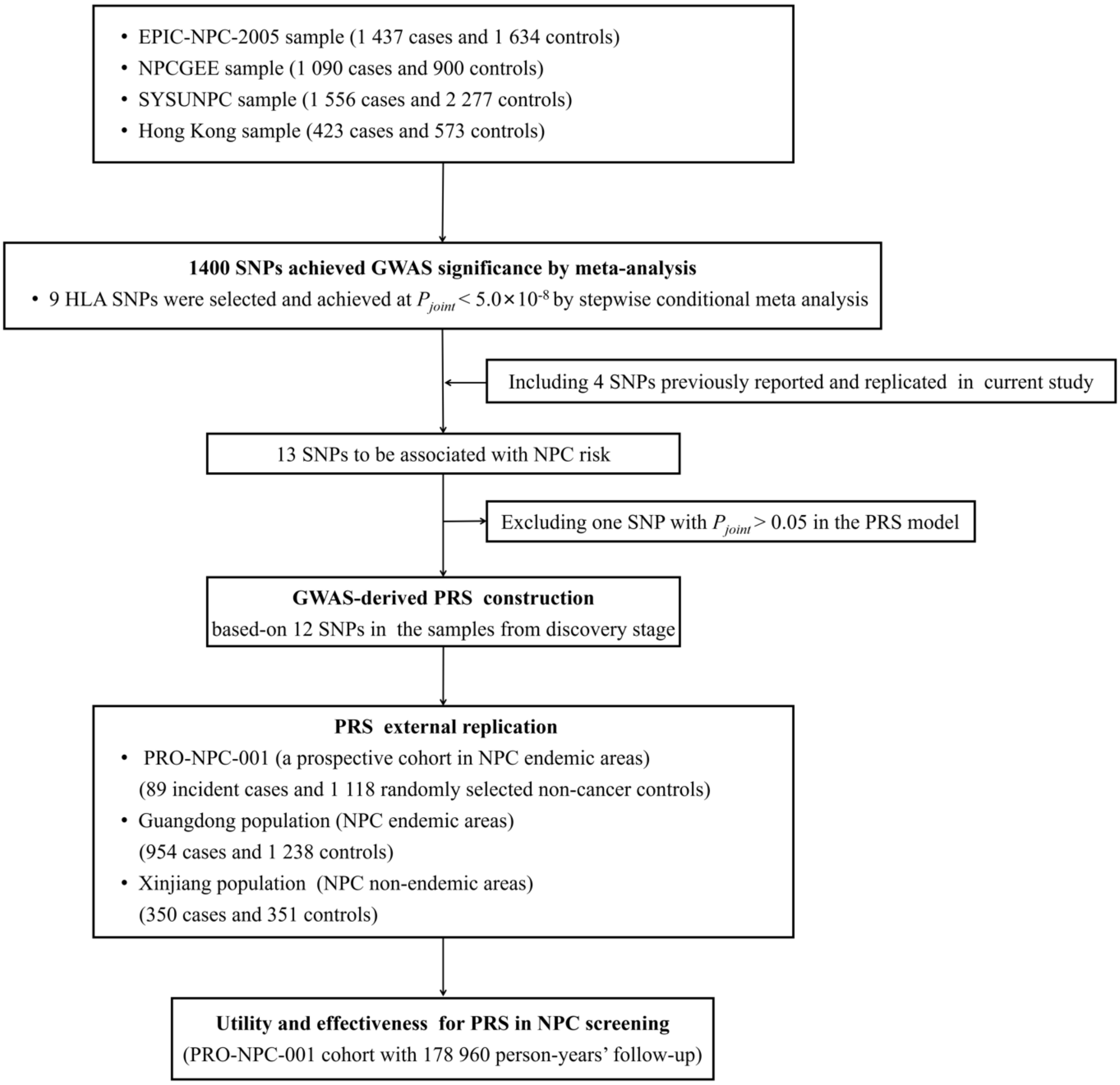

Supplementary Tables

Supplementary Table 1. Nasopharyngeal carcinoma risk signals previously reported at genome-wide significance

CHR	SNP	BP	Locus	Nearest Gene	Risk Allele	OR	*P*	*P* *	Reference(s) (first author and year)	PubMed ID
3	rs189897	37518545	3p21	ITGA9	A	1.02	8.30E-01	6.85E-08	Ching Ching Ng et al. 2009	19478819
3	rs6774494	169082633	3q26.2	MECOM	G	0.84	7.41E-07	1.00E-08	Bei JX et al. 2010	20512145
5	rs31489	1342714	5p15.33	CLPTM1L	A	0.77	3.79E-10	6.00E-13	Bei JX et al. 2016	26545403
5	rs401681	1322087	5p15.33	CLPTM1L	T	0.82	4.88E-08	3.00E-14	Cui Q et al. 2016	27436580
6	rs2517713	29950322	6p22.1	HLA-A	G	0.55	2.04E-55	4.00E-20	Tse KP et al. 2009	19664746
6	rs29232	29643654	6p22.1	GABBR1	T	1.53	2.09E-35	9.00E-17	Tse KP et al. 2009	19664746
6	rs2267633	29570841	6p22.1	*GABBR1*	G	0.63	5.88E-29	1.28E-09	Tang MZ et al. 2012	23209447
6	rs2076483	29571545	6p22.1	*GABBR1*	G	0.63	5.88E-29	1.49E-09	Tang MZ et al. 2012	23209447
6	rs29230	29576393	6p22.1	*GABBR1*	G	0.62	1.77E-29	4.77E-09	Tang MZ et al. 2012	23209447
6	rs3129055	29702484	6p22.1	ZFP57	G	1.38	6.36E-19	7.00E-11	Tse KP et al. 2009	19664746
6	rs417162	29916505	6p22.1	*HLA-A;HCG9*	C	0.55	5.85E-55	1.05E-11	Tang MZ et al. 2012	23209447
6	rs7747253	29919779	6p22.1	*HLA-A;HCG9*	C	0.53	8.88E-64	2.08E-09	Yoon-Ming Chin et al. 2015	24947555
6	rs2975042	29920536	6p22.1	*HLA-A;HCG9*	G	0.56	3.30E-52	1.60E-19	Tang MZ et al. 2012	23209447
6	rs9260734	29932666	6p22.1	*HLA-A;HCG9*	A	0.57	3.40E-46	6.77E-18	Tang MZ et al. 2012	23209447
6	rs3869062	29934891	6p22.1	*HLA-A;HCG9*	G	0.59	2.67E-39	8.68E-16	Tang MZ et al. 2012	23209447
6	rs2860580	29938914	6p22.1	HLA-A	A	0.56	2.17E-53	3.00E-31	Bei JX et al. 2010	20512145
6	rs5009448	29940488	6p22.1	*HLA-A;HCG9*	T	0.61	5.13E-41	1.30E-15	Tang MZ et al. 2012	23209447
6	rs2893999	29943832	6p22.1	*HCG9*	C	0.59	1.49E-39	2.61E-09	Yoon-Ming Chin et al. 2015	24947555
6	rs16896923	30000687	6p22.1	*ZNRD1ASP*	C	0.63	1.79E-26	2.49E-10	Yoon-Ming Chin et al. 2015	24947555
6	rs2894207	31295974	6p21.33	HLA-B,HLA-C	C	0.51	1.15E-40	2.00E-19	Bei JX et al. 2010	20512145
6	rs28421666	32624960	6p21.32	HLA-DQA1	G	0.68	2.22E-12	1.00E-10	Bei JX et al. 2010	20512145
9	rs1412829	22043926	9p21.3	CDKN2B-AS1	G	0.72	6.79E-09	3.00E-08	Bei JX et al. 2016	26545403
13	rs9510787	24205195	13q12.12	TNFRSF19	G	1.16	1.96E-05	2.00E-09	Bei JX et al. 2010	20512145
13	rs1572072	23553071	13q12.12	TNFRSF19	T	0.92	3.70E-02	1.00E-08	Bei JX et al. 2010	20512145
16	rs6498114	10870261	16p13.13	CIITA	A	0.88	1.25E-04	4.00E-09	Cui Q et al. 2016	27436580

CHR, chromosome; SNP, rsID on NCBI dbSNP Build 150; BP, base pair position (hg19); OR, odds ratio of the risk allele.
*P for published GWAS index variant is the P-value observed in the cited GWAS study.

Supplementary Table 2. Stepwise conditional analysis found 9 SNPs associated with NPC risk in HLA region, and 6 of them (marked by *) remained statistical significance after adjusting the previously reported HLA SNPs

CHR	SNP	BP	A1	Nearest Gene	Unconditional Analysis ^		Conditional Analysis #		Excluding PCs $	
					OR (95% CI)	*P*	OR (95% CI)	$P_{conditional}$	OR (95% CI)	$P_{conditional}$
6	rs3131875 *	29666111	T	ZFP57/HLA-F	0.75 (0.70-0.81)	4.15E-15	1.97 (1.78-2.18)	1.66E-39	1.97(1.78-2.17)	3.19E-41
6	rs1611163 *	29792008	T	HLA-G upstream	0.47 (0.44-0.52)	4.83E-67	0.54 (0.49-0.60)	1.39E-32	0.56(0.50-0.61)	2.02E-31
6	rs9357092 *	29984252	A	ZNR1ASP	1.51 (1.41-1.62)	7.76E-30	2.04 (1.85-2.25)	8.74E-48	2.00(1.82-2.20)	7.32E-48
6	rs9261506	30112623	A	TRIM40	0.76 (0.69-0.85)	2.20E-07	0.29 (0.23-0.36)	4.14E-28	0.29(0.23-0.36)	1.23E-28
6	rs9261567	30185244	C	TRIM26/HCG17	0.78 (0.71-0.86)	5.44E-07	4.37 (3.51-5.43)	1.03E-39	4.15(3.35-5.15)	1.86E-38
6	rs2251830	31016978	A	MUC22/HCG22	1.20 (1.11-1.29)	1.61E-06	1.71 (1.55-1.88)	3.00E-28	1.70(1.55-1.87)	6.17E-29
6	rs2596506 *	31318553	T	HLA-B downstream	0.70 (0.64-0.75)	6.31E-20	0.54 (0.49-0.59)	4.67E-37	0.53(0.48-0.58)	6.16E-40
6	rs2844484 *	31536224	A	NFKBIL1/LTA	0.83 (0.77-0.89)	1.06E-06	0.64 (0.59-0.70)	5.46E-24	0.64(0.59-0.70)	3.22E-25
6	rs9268644 *	32408044	A	HLA-DRA	0.66 (0.59-0.72)	1.04E-16	0.65 (0.58-0.73)	1.61E-14	0.66(0.59-0.73)	1.86E-14

CHR, chromosome; SNP, rsID on NCBI dbSNP build 150; BP, physical position of SNP in hg19; A1, represents the minor allele; MAF, minor allele frequency in all the samples; OR, odds ratio estimates for the minor allele; CI, confidence interval. Multivariate analysis was performed to jointly estimate the effect of each selected variants. Due to the extensive and complex LD structure of the MHC region, we performed the analysis overall 29-34 Mb region on chromosome 6.

* The SNPs that have additional contribution to NPC risk after adjusting the effects of the five reported SNPs, rs2076483, rs5009448, rs2894207, rs28421666 and rs29232 with $P < 5.0\times10^{-8}$.

^ The OR and *P* were derived from meta-GWAS analysis.

Stepwise conditional meta-analysis by adjusting age, sex and PCs.

$ Stepwise conditional meta-analysis by adjusting age and sex.

Supplementary Table 3. The odds ratio and the risk allele frequencies of the SNPs in the model for cumulative NPC incidence risk estimation

SNP	CHR	BP	Nearest Gene	A1	Frequency	OR_{joint} (95%CI)
rs2106123	3	169057657	MECOM	G	0.35	0.84 (0.78 - 0.90)
rs31489	5	1342714	CLPTMIL	A	0.23	0.77 (0.70 - 0.85)
rs3131875	6	29666111	ZFP57/HLA-F	T	0.40	1.23 (1.15 - 1.32)
rs1611163	6	29792008	HLA-G upstream	T	0.31	0.61 (0.52 - 0.69)
rs9357092	6	29984252	ZNR1ASP	A	0.34	1.26 (1.18 - 1.33)
rs9261506	6	30112623	TRIM40	A	0.14	0.84 (0.74 - 0.93)
rs2251830	6	31016978	MUC22/HCG22	A	0.29	1.30 (1.23 - 1.38)
rs2596506	6	31318553	HLA-B downstream	T	0.39	0.70 (0.63 - 0.77)
rs2844484	6	31536224	LTA/NFKBIL1	A	0.27	0.87 (0.80 - 0.94)
rs9268644	6	32408044	HLA-DRA	A	0.16	0.63 (0.54 - 0.72)
rs6475604	9	22052734	CDKN2B	T	0.12	0.74 (0.64 - 0.84)
rs9507124	13	24208927	TNFRSF19	C	0.35	1.16 (1.10 - 1.21)

CHR, chromosome; BP, base pair position (hg19), SNP, rsID on NCBI dbSNP build 150; A1, minor allele; OR_{joint} were the odds ratio and P values by multivariate analysis.

Supplementary Table 4. Incremental contribution of polygenic score to NPC detection compared with family history in the EPI-NPC-2005 samples, the NPCGEE samples, and the combined samples

Samples	Model	Crude Λ (bits) [#]	Model-Based Λ (bits) [#]	Incremental	Difference in test log likelihood (nats) [*]	Difference in log likelihood after Recalibration (nats) [*]
EPI-NPC-2005	family history	0.12	0.11	-	-	-
	PRS+ family history	0.35	0.33	0.22	101	102
NPCGEE	family history	0.13	0.11	-	-	-
	PRS+ family history	0.31	0.27	0.16	42	43
Combined	family history	0.12	0.12	-	-	-
	PRS+ family history	0.33	0.32	0.2	148	148

Information for discrimination Λ is measured in bits.

*An increment in the test log-likelihood ≥6.9 natural log units are asymptotically equivalent to a p value <0.005, therefore all results are highly significant.

Supplementary Table 5. The hazard ratios between PRS and NPC risk in PRO-NPC-001 cohort

PRS	Controls (%)	Cases (%)	HR (95%CI)	*P*
<P20	182 (97.85)	4 (2.15)	1.00 (reference)	/
P20-P30	126 (93.33)	9 (6.67)	3.06 (0.94 – 9.97)	0.063
P30-P40	126 (94.03)	8 (5.97)	2.68 (0.80 – 9.05)	0.111
P40-P50	131 (96.32)	5 (3.68)	1.68 (0.45 – 6.24)	0.442
P50-P60	109 (95.61)	5 (4.39)	1.97 (0.53 – 7.42)	0.314
P60-P70	144 (92.31)	12 (7.69)	3.54 (1.13 -11.04)	0.030
P70-P80	99 (91.67)	9 (8.33)	3.56 (1.10 – 11.54)	0.034
P80-P90	107 (88.43)	14 (11.57)	5.25 (1.71 – 16.09)	0.004
≥P90	94 (80.34)	23 (19.66)	9.17 (3.19 – 26.35)	3.89E-05

HR, hazard ratio. The hazard ratios and P-values were calculated by Cox regression adjusted by age and sex.

Supplementary Table 6. The number and proportion of missed diagnosis NPC cases who were defined as low-risk by EBV tests in PRO-NPC-001 cohort in different PRS subgroup *

PRS	Number	%
<P10	0	0
P10-P20	0	0
P20-P30	0	0
P30-P40	0	0
P40-P50	1	5.26
P50-P60	1	5.26
P60-P70	4	21.05
P70-P80	3	10.53
P80-P90	3	15.79
≥P90	8	42.11
All	19	100.00

* With a median follow up of 7.33 years (IQR 3.20-7.87), 19 incident NPC cases were identified among 27 657 EBV seronegative individuals.

Supplementary Table 7. Demographic information and genotyping information for the six samples

Study	Cases (patients with NPC)					Controls (Subjects without cancer)				
	N	Males	Females	Age (IQR)	Platform (n)	N	Males	Females	Age (IQR)	Platform (n)
EPIC-NPC-2005	1614	1181	433	45(38-54)	Human610-Quadv1	1819	1291	528	49(42-57)	Human610-Quadv1; GSAMD-24V1
NPCGEE	1098	804	294	47(41-56)	Illumina Infinium Global Screening Array -24V1	991	721	270	46(40-55)	Illumina Infinium Global Screening Array -24V1
SYSUNPC	1617	1188	429	43(37-51)	Illumina Infinium Global Screening Array -24V1	2610	1631	979	61(58-68)	Illumina Infinium Global Screening Array -24V1
Hong Kong	426	323	103	53(45-60)	Infinium Asian Screening Array	573	349	224	51(40-58)	Infinium Asian Screening Array
Guangdong	954	601	353	51(43-57)	Agena MassARRAY	1238	878	360	39(34-47)	Agena MassARRAY
Xinjiang	350	248	102	48(42-57)	Agena MassARRAY	351	144	207	36(29-47)	Agena MassARRAY

IQR: interquartile range

Supplementary Table 8. Demographic characteristics of 29 413 participants in PRO-NPC-001 cohort

Variables	Anti-EBV (+) group	Anti-EBV (-) group	Total
Sex			
Male	989	13 910	14 899
Female	767	13 747	14 514
Age, years			
Median (IQR)	48 (41 - 54)	46 (40 - 53)	46 (40 - 53)
30-34	120	1 956	2 076
35-39	235	4 748	4 983
40-44	298	5 717	6 015
44-49	315	5 414	5 729
50-54	363	4 546	4 909
55-59	425	5 276	5 701
Follow-up, person-years			
Total person-years	9 460	169 499	178 960
Number of incidence NPC	70	19	89

IQR: interquartile range

Supplementary Table 9. The imputation quality score for the six novel SNPs in the 4 GWAS studies

SNP	EPIC-NPC-2005	NPCGEE	SYSUNPC	HK
rs3131875	0.73	0.77	0.78	0.64
rs1611163	0.79	0.80	0.80	0.84
rs9357092	0.94	0.75	0.75	/
rs2596506	0.80	0.92	0.94	0.94
rs2844484	0.81	0.78	0.84	0.99
rs9268644	0.96	0.94	0.94	0.99

Supplementary Table 10. Summary statistics of the samples and imputation SNPs in the discovery stage

Study	Before quality control			After quality control		
	SNPs	Cases	Controls	SNPs	Cases	Controls
EPIC-NPC-2005	700 078	1 614	1 819	4 835 244	1 437	1 634
NPCGEE	700 078	1 098	991	5 066 825	1 090	900
SYSUNPC	700 078	1 617	2 610	5 099 736	1 556	2 277
Hong Kong	657 060	426	573	4 902 485	423	573
Total		4 755	5 993		4 506	5 384

Supplemental Table 11 The Consistency of SNP genotypes between GWAS genotyping and sanger sequencing.

SNP	Number of tests	Number of consistent tests	Consistent rate
rs1611163 *	47	47	100%
rs3131875 *	47	47	100%
rs9357092 *	47	47	100%
rs9261506 *	47	47	100%
rs2844484 *	47	47	100%
rs9268644 *	47	47	100%
rs2251830	47	47	100%
rs9261567	47	47	100%
rs2596506	47	47	100%
rs1867277	109	108	99.1%
rs226241	107	104	97.2%
Total test	639	635	99.4%

* The novel identified SNPs.

Supplementary Reference

1. Jia WH, *et al.* Traditional Cantonese diet and nasopharyngeal carcinoma risk: a large-scale case-control study in Guangdong, China. *BMC cancer* 10, 446 (2010).

2. Xu FH, *et al.* An Epidemiological and Molecular Study of the Relationship Between Smoking, Risk of Nasopharyngeal Carcinoma, and EpsteinBarr Virus Activation. *Jnci-J Natl Cancer I* 104, 1396-1410 (2012).

3. Bei JX, *et al.* A genome-wide association study of nasopharyngeal carcinoma identifies three new susceptibility loci. *Nature genetics* 42, 599-603 (2010).

4. Ye W, *et al.* Development of a population-based cancer case-control study in southern china. *Oncotarget* 8, 87073-87085 (2017).

5. Lv JW, *et al.* Hepatitis B virus screening and reactivation and management of patients with nasopharyngeal carcinoma: A large-scale, big-data intelligence platform-based analysis from an endemic area. *Cancer* 123, 3540-3549 (2017).

6. Dai J, *et al.* Identification of risk loci and a polygenic risk score for lung cancer: a large-scale prospective cohort study in Chinese populations. *The Lancet Respiratory medicine* 7, 881-891 (2019).

7. Mai ZM, *et al.* Test-retest reliability of a computer-assisted self-administered questionnaire on early life exposure in a nasopharyngeal carcinoma case-control study. *Scientific reports* 8, 7052 (2018).

8. Mai ZM, *et al.* Milk Consumption Across Life Periods in Relation to Lower Risk of Nasopharyngeal Carcinoma: A Multicentre Case-Control Study. *Frontiers in oncology* 9, 253 (2019).

9. Liu Z, *et al.* Two Epstein-Barr virus-related serologic antibody tests in nasopharyngeal carcinoma screening: results from the initial phase of a cluster randomized controlled trial in Southern China. *American journal of epidemiology* 177, 242-250 (2013).

10. Ji MF, *et al.* Incidence and mortality of nasopharyngeal carcinoma: interim analysis of a cluster randomized controlled screening trial (PRO-NPC-001) in southern China. *Ann Oncol* 30, 1630-1637 (2019).

11. Purcell S, *et al.* PLINK: a tool set for whole-genome association and population-based linkage analyses. *American journal of human genetics* 81, 559-575 (2007).

12. Price AL, Patterson NJ, Plenge RM, Weinblatt ME, Shadick NA, Reich D. Principal components analysis corrects for stratification in genome-wide association studies. *Nature genetics* 38, 904-909 (2006).

13. Delaneau O, Marchini J, Zagury JF. A linear complexity phasing method for thousands of genomes. *Nature methods* 9, 179-181 (2011).

14. Howie BN, Donnelly P, Marchini J. A flexible and accurate genotype imputation method for the next generation of genome-wide association studies. *PLoS genetics* 5, e1000529 (2009).

15. Jia X, *et al.* Imputing amino acid polymorphisms in human leukocyte antigens. *PloS one* 8, e64683 (2013).

16. Zhou F, *et al.* Deep sequencing of the MHC region in the Chinese population contributes to studies of complex disease. *Nature genetics* 48, 740-746 (2016).

17. Willer CJ, *et al.* Newly identified loci that influence lipid concentrations and risk of coronary artery disease. *Nature genetics* 40, 161-169 (2008).

18. Sanna S, *et al.* Common variants in the GDF5-UQCC region are associated with variation in human height. *Nature genetics* 40, 198-203 (2008).
