# Supplementary material for: A polygenic risk score for nasopharyngeal carcinoma shows potential for risk stratification and personalized screening
Source: Nat Commun. 2022 Apr 12;13:1966. doi: 10.1038/s41467-022-29570-4 (PMC9005522; doi:10.1038/s41467-022-29570-4)
Supplement: Supplementary file 2 — Description of Additional Supplementary Files [file 41467_2022_29570_MOESM2_ESM.pdf]

File Name: Supplementary Data 1

Description: Age-specific NPC incidence of Zhongshan city of China, derived from International Agency Research on Cancer Incidence in Five Continents Volume XI.
